# Interpreting Emotions From Women With Covered Faces: A Comparison Between a Middle Eastern and Western-European Sample

**DOI:** 10.3389/fpsyg.2021.620632

**Published:** 2021-05-07

**Authors:** Mariska E. Kret, Angela T. Maitner, Agneta H. Fischer

**Affiliations:** ^1^Cognitive Psychology Unit, Institute of Psychology, Leiden University, Leiden, Netherlands; ^2^Leiden Institute for Brain and Cognition (LIBC), Leiden, Netherlands; ^3^Department of International Studies, American University of Sharjah, Sharjah, United Arab Emirates; ^4^Social Psychology Department, University of Amsterdam, Amsterdam, Netherlands

**Keywords:** emotion recognition, face coverage, veiled faces, cross-cultural comparison, interpretation bias

## Abstract

While new regulations obligate or recommend people to wear medical masks at public places to prevent further spread of the Covid-19 virus, there are still open questions as to what face coverage does to social emotional communication. Previous research on the effects of wearing veils or face-covering niqabs showed that covering of the mouth led to the attribution of negative emotions and to the perception of less intense positive emotions. The current study compares a sample from the Netherlands with a sample from the United Arab Emirates on their perception of emotions from faces covered by a niqab, censoring black bars, or uncovered faces. The results show that covering the mouth area leads to greater anxiety in participants in both countries. Furthermore, although participants did not report greater decoding difficulties for faces that were covered as compared to fully visible, results show that face coverage did influence emotion perception. Specifically, happiness and anger were perceived as being less intense. Further, face coverage by a niqab, as compared to black bars, yielded lower emotional intensity ratings. We conclude that face coverage in particular can modulate the perception of emotions, but that affective contextual cues may play a role as well.

## Introduction

We perceive emotions on the basis of expressive and contextual cues provided by the people around us (Russell and Barrett, 1999; Barrett, 2006). With regard to displays of emotion, facial expressions have most often been examined, as they are regarded as crucial in differentiating different types of emotion (Ekman, 1992, 1993). There are situations, however, where emotional expressions are hardly visible. In the current Covid-19 crisis for example, medical masks, obligated in supermarkets, public transport and other places, render the mouth invisible. Other examples can be found in the context of sports (helmets), in winter, when scarves are occluding the face, or when women wear a niqab which covers much of the face, leaving only the eyes visible. This latter example has gained attention in political debates in many Western countries where the question of banning face covering veils such as niqabs has been raised (Moors, 2009). An important motive for advocating such a prohibition is that it would impair the recognition of emotion, and thus hinders interpersonal communication (Van der Noll, 2010). Indeed, previous research has shown that Western participants process emotions differently when the face is covered than when it is fully visible. Specifically, coverage of the mouth region interferes with the processing of positive emotions such as happiness, and results in a more negative interpretation of the observed emotion (Fischer et al., 2012; Kret and de Gelder, 2012; Everett et al., 2014; Kret and Fischer, 2018; Liedtke et al., 2018). These studies provide evidence for the idea that blocking expressive cues on the face interferes with emotion perception. However, whether or not it is face coverage in general that promotes these deficits, or whether it is specifically Islamic contextual cues that yield certain biases and color emotion processing, is still undetermined due to mixed evidence in previous literature. The current study aims to shed light on this open question. In addition, while the majority of studies looking into this topic examined the effects of niqabs in participants from cultures where Islam is a minority religion, the current study compares participants from two different cultures varying in their levels of exposure to symbols of Islamic identity.

Our aim, therefore, was to examine whether biases in perceived emotion of women wearing a niqab result from a lack of expressive cues or the (mis)use of contextual cues. To that end, we compared participants from a Western country (the Netherlands) and a Middle Eastern country (the United Arab Emirates), where wearing niqabs or covering the face is, relatively speaking, less surprising. If Dutch participants experience more anxiety in reaction to covered faces than participants from the United Arab Emirates (UAE), this could subsequently affect the perception of emotions in the Dutch sample more than in the UAE sample, but only when perceiving a woman wearing a niqab and not a women whose face is uncovered or covered with black bars. If, on the other hand, emotion perception in covered faces can be solely explained by the lack of expressive cues, particularly the mouth, then the perception of emotions in covered faces (compared to non-covered faces) should not be different in the two samples. Moreover, participants’ emotion perception of a woman in a niqab should then be similar to perceptions of her when her face is occluded by black bars.

### Lack of Expressive Cues or Anxiety?

An abundant number of studies have shown that individuals are able to recognize discrete emotions (such as anger, contempt, disgust, fear, sadness, surprise, and happiness) from actors posing the associated expressions (Ekman, 1993; Keltner, 1995; Matsumoto et al., 2008). The recognition of these emotions is based on a number of specific muscle activations in either the upper, middle or lower part of the face. This implies that covering a specific part of the face would have more impact on the recognizability of some emotions than of others (e.g., Eisenbarth and Alpers, 2011). Wearing a niqab – which only leaves the eyes visible – would have the strongest effects on the perception of emotions that are prototypically displayed in the lower part of the face, such as is the case with smiling. In an earlier study examining the effects of decoding emotions in women wearing niqab, Fischer et al. (2012) exposed participants to short videos without sound, in which they watched women telling a story while displaying anger, shame or happiness, either with a covered face (niqab or censoring black bars) or an uncovered face. They found that perceiving happiness was most affected by face coverage, which can be explained by the fact that a smile cannot be seen when the lower part of the face is covered. The perception of anger was least affected, in line with the expectation that negative emotion displays that typically involve contraction of the *Corrugator Supercilii* (e.g., anger, fear, disgust, which are accompanied by frowning) could still be recognized if only the lower part of the face is covered. Shame, which does not involve the *Corrugator Supercilii*, but is typically characterized by an averted gaze (Keltner, 1995), or by the head being tilted downward, was affected by face covering (regardless of the type of covering), showing that perceivers infer more intense shame from covered shame displays. The fact that shame was also affected by face covering was explained by the fact that a shame display is a relatively ambiguous expression, which is not recognized as well as other negative emotions (e.g., Hawk et al., 2009). However, it is still possible that these effects were primarily driven by the niqab condition. Therefore, an alternative explanation could be that observers expect women wearing a niqab to feel more shame and less happiness, due to the contextual cue (niqab) being associated with the stereotype of a suppressed woman.

Indeed, when expressive cues are partly absent and emotions are thus more difficult to detect, observers are likely to make use of other cues when asked which emotion they recognize. There is growing evidence that emotion perception is influenced by contextual cues such as the emotional scene (e.g., Righart and de Gelder, 2008; Aviezer et al., 2009; Kret and de Gelder, 2010; Kret et al., 2013a), availability of emotion words (Lindquist et al., 2006), situational information (e.g., Carroll and Russell, 1996) or by conflicting information from within the observed person, such as his or her identity (e.g., Hugenberg, 2005; Hugenberg and Sczesny, 2006; Bijlstra et al., 2010) or body language (Meeren et al., 2005; Kret et al., 2013b). A general conclusion that we can draw from these studies is that the recognition of emotions is facilitated when congruent contextual information is provided.

It is obvious that wearing a niqab is a salient contextual cue, at least in Western countries. It is a symbol of Islamic identity (e.g., Moors, 2009), and may trigger negative feelings in Western individuals (e.g., Islam and Hewstone, 1993). These negative feelings may in turn bias the perceived intensity of negative emotions. Previous studies (Everett et al., 2014) have indeed shown that neutral looking women wearing a scarf (i.e., hijab) or niqab elicit negative reactions, compared to women without any veil. Research on social categorization and emotion recognition has further demonstrated that faces belonging to a specific social category trigger negative evaluations that facilitate valence-congruent emotion recognition (Hugenberg and Bodenhausen, 2004; Hugenberg, 2005). Moreover, the content of stereotypes may also affect the recognition of specific emotions. For example, anger is recognized faster in an individual that belongs to a group that is stereotypically regarded as aggressive (Hess et al., 2005; Bijlstra et al., 2010), whereas fear is recognized more readily in women wearing niqabs (Kret and de Gelder, 2012; Liedtke et al., 2018).

Yet, whereas Fischer et al. (2012) found differences in emotion perception when the face was covered versus fully visible, hardly any differences were found between the two different face covering conditions (niqab and black bars). This suggests that contextual cues alone did not affect the inference of emotional intensity from a face and that the findings could be explained by the mere covering of the face. Still, other studies have shown that contextual cues can lead to biased emotion perception that is more congruent with contextual cues. Some studies on the perception of emotions in faces covered to some extent by a veil have indeed suggested that specific contextual cues may elicit negative feelings in observers, which in turn affect how emotions are perceived in the face. For instance, it has been shown that angry, fearful, happy and sad facial expressions were interpreted differently, depending on the type of headgear surrounding the face (Kret and de Gelder, 2012). Specifically, these facial expressions were presented for less than 100 ms and participants had to press the corresponding emotion label as fast and accurately as they could. In half of the trials, the faces were covered by an Islamic veil [niqab or hijab (which leaves the mouth visible and only covers the hair and contours of the face)], in the other half by a cap or cap and a scarf, covering the exact same face areas. The same paradigm was used in two follow-up studies. In one, potential modulating effects of oxytocin were investigated (Kret and Fischer, 2018), in the other depression (Liedtke et al., 2018). The key finding that was consistent across these three studies is that the recognition of happiness was impaired from the faces that were specifically covered with a niqab (and not a cap and scarf of the type people wear in winter times) (Kret and de Gelder, 2012; Kret and Fischer, 2018; Liedtke et al., 2018). Niqabs may thus serve as a contextual cue and therefore influences emotion perception. Previous research has shown that niqabs may elicit intergroup anxiety in individuals in Western countries, where wearing a niqab is uncommon (Stephan and Stephan, 1985; Stephan, 2014). Possibly, when expressive cues are missing, contextual cues play a more important role in interpreting emotions.

The present study further examined the role of expressive versus contextual cues by extending previous research in two ways. First of all, we included samples from two countries that differ in the salience and frequency of exposure to Islamic symbols, such as a niqab (e.g., Moors, 2009), Netherlands and the UAE. Islamic symbols may be more likely to trigger negative stereotypes and intergroup anxiety in Western, non-Muslim individuals (e.g., Islam and Hewstone, 1993) than in individuals living in an Islamic country. Therefore, if contextual cues alone explain biases in the perception of emotions expressed by women wearing a niqab, then we should expect differences across the two samples in the perception of emotions expressed by women wearing a niqab, but not when women are uncovered, or when their faces are occluded by other means. If a lack of expressive cues explains biases in the perception of emotions from faces covered by a niqab, then we should see a similar pattern of emotional attributions across the two samples, and similar attributions of emotion to women whose faces are covered by either a niqab or black bars.

We included two additional measures. First, we included a measure that taps into intergroup anxiety (Stephan and Stephan, 1985). Muslims are more likely to be considered outgroup members to Dutch participants than to UAE participants. The contextual cue of the niqab, then, may elicit more intergroup anxiety in Dutch than UAE participants (Stephan and Stephan, 1985; Islam and Hewstone, 1993), leading to the fact that Dutch participants may feel less at ease when interacting with a woman wearing a niqab (see also Hewstone et al., 2002). We expected that this negative feeling would increase the perceived intensity of negative emotions and decrease the perceived intensity of positive emotions, if contextual cues play a strong role in the perception of emotion (but see Kret and de Gelder, 2012). Second, we asked whether participants found it difficult to recognize emotions in the targets (decoding difficulty), which served as a control question for the fact that the two samples may have different experiences with such tasks. We expected no differences between the two groups of respondents on this measure.

In sum, we tested the following hypotheses: (1) *Expressive Cues Hypothesis:* Covering the face with anything (black bars or niqab) leads to the perception of less intense happiness, more intense shame, and similar amounts of anger in both samples; (2) *Contextual Cues Hypothesis:* Covering the face with a niqab (but not black bars) specifically leads to the perception of less intense happiness and more intense shame in the Dutch but not the UAE sample.

## Present Study

In the current study participants from the Netherlands and from the UAE were presented with three short film clips displaying an expression of emotion. Participants first saw a neutral clip, followed by women expressing anger, happiness and shame (in random order). The women in the clips were shown in one of three different face conditions: one in which the whole face is visible (full face condition), one in which the woman wears a niqab (niqab condition), and one condition in which we put censoring black bars across the top and bottom of the face (black bars condition), after which only the eyes and eyebrows were visible. We added this condition in order to compare different contextual cues. In contrast to the niqab, the black bars condition creates equal decoding difficulty, but is affectively neutral. Although a face covered by black bars could be perceived as negatively valenced, a black bar in and on itself is neither positive nor negative, making it a neutral context cue.

**FIGURE 1:**
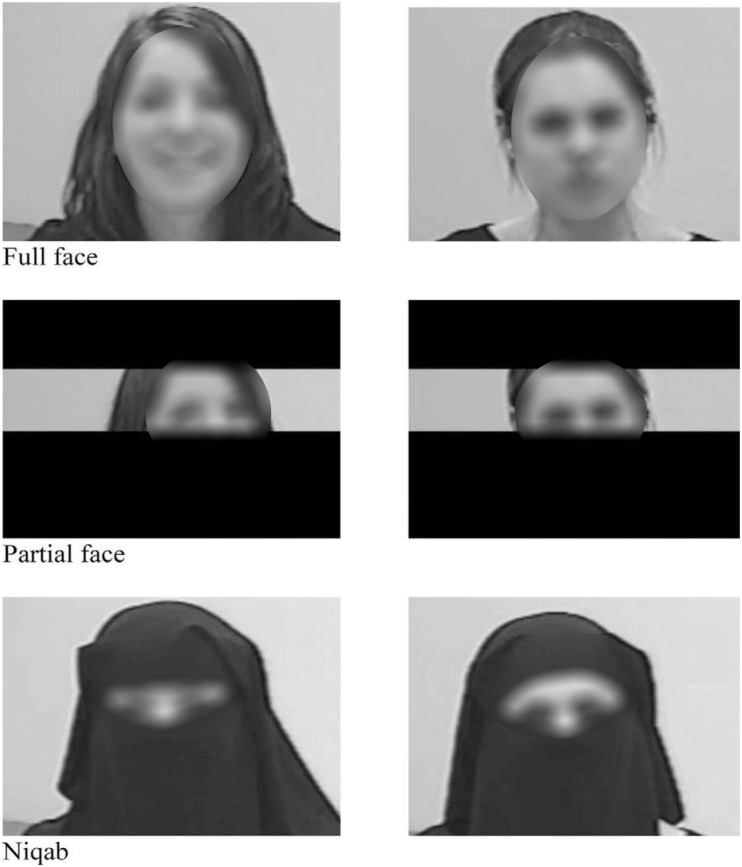
Stills from emotion videos, full face, covered face (black bar and niqab).

The short film fragments were about 1 min long and showed female models telling an emotional story while expressing anger, happiness or shame (see Figure 1 for stills from the video clips). The participants watched the film clips without sound, in order to have only visual cues available. We selected these three emotions for two reasons. First, they involve movements of different parts of the face, and we are thus able to examine whether the perception of happiness is more impaired than the perception of anger and shame by masking the mouth. A second reason to focus on these three emotions is that they are differently related to stereotypical expectations about Muslim women.

In short, after watching a neutral video, participants watched three different, muted, videos of a woman displaying happiness, anger and shame while telling a story. The women were either shown without niqab (full face), with niqab, or with their face partly covered by black bars.

## Method

### Participants

We collected data from respondents studying at a Dutch University and at a university in the UAE. The Dutch sample consisted of 117 students (*M*_*age*_ = 21.9 years, *SD* = 4.65, 35 males and 72 females), of which 106 had a Dutch and 11 had another Western European nationality (e.g., German and Belgian). The UAE sample consisted of 200 students: 57 students from countries in the Gulf Cooperation Council (GCC: Bahrain, Qatar, Kuwait, Oman, Saudi Arabia and the UAE); 86 from non-GCC Arab countries (e.g., Egypt, Syria, Jordan, Palestine), and 48 respondents from non-Arab countries (e.g., India and Pakistan). They all received course credits for participation. We aimed to recruit a sample of 100 participants per location, but the final number was a result of lab space availability and signups during that period. We would like to stress that our sample size is much larger than previous studies studying emotion perception in the context of Islamic cues (Fischer et al., 2012; Kret and de Gelder, 2012; Kret and Fischer, 2018). All data were collected at the respective university and all participants were randomly assigned to one of the three face conditions.

### Design

The study has 2 between-subjects factors: Face Coverage (3: full face, niqab, black bars) and Country (Netherlands, UAE). Emotional Display (happiness, anger, shame) was manipulated within-subjects.

### Dependent Measures

**Emotion perception.** After each emotion video, we asked to what extent participants thought the woman experienced anger, shame, happiness, distress, disappointment, and sadness (1 = *not at all*; 7 = *very much*)^1^. We included more than the three target emotions, in order to make the task less easy.

**Decoding Difficulty.** After all three videos, we asked how confident they had been regarding their perception of emotions (1 = *very uncertain*; 7 = *very certain*) and how difficult they had found the perception of emotion (1 = *difficult*; 7 = *easy*). As these two variables correlated (*r* = 0.42, *p* < 0.001), we computed a composite scale.

**Anxiety.** After all three videos, we also asked how they would evaluate future interaction with this woman (1 item: 1 = *very negative*; 7 = *very positive*, reverse scored) and to what extent they would feel at ease while talking to her (1 item; 1 = *feeling not at ease*; 7 = *feeling at ease*, reverse scored); again the two items were correlated (*r* = 0.77, *p* < 0.001) and combined to create one scale (i.e., higher scores refers to more anxiety).

**Stimulus materials**. In order to control for idiosyncratic effects of target woman, we had three different female models tell the same stories in each emotion condition. All models had dark eyes and hair, so that each of them could be perceived stereotypically as either of Arab heritage or not, and that all women could be seen as possibly local in both cultural contexts. Which model was seen varied between participants. We wrote three different texts that would evoke the three emotions. The women were asked whether they recognized the situation in the story and whether they felt they could empathize with the person in the story and tell the story as if it had happened to them. The storytelling was rehearsed and videotaped. Each woman first told a neutral story without niqab, then the same story with niqab in order to minimize differences between face coverage. The same procedure was followed for the three emotional stories. Length of the films was standardized per emotion, so that all anger, happiness, and shame videos were of equal length. The videos lasted about 1 min and 20 s. The black bars condition was edited later from the full face videos.

Each neutral introduction consisted of the woman sharing her name, age, and address and some neutral facts about her family and education. The “anger story” was about a teacher who treated them unfairly, the “happiness story” was about having passed a driving exam and getting a driver’s license, and the “shame story” was about congratulating an acquaintance about her pregnancy and finding out that she had only gained weight. The women were instructed to show specific facial expressions while telling the emotional stories. To express anger, they were instructed to use the *Corrugator Supercilii* muscle (frown). For shame, they were instructed to avert their gaze, and to look down. For happiness, they were instructed to smile (*Zygomaticus Major*).

### Procedure

After arriving, Dutch participants were told that the experiment aimed at increasing our insight into conveying emotions through video images, and that they were going to see a number of videos in which a woman would tell a personal story that they could not hear, because they had to focus on what they saw. They read an information letter and signed informed consent, and were then lead to a test cubicle. Subsequently, they were presented with short videos in which they saw the same woman telling a neutral story, and subsequently three different emotional stories presented in random order. After each video, they completed an emotion recognition measure, and completed the decoding difficulty and anxiety measures once all videos were completed. Finally, participants were asked several exit questions, after which they received compensation for participation and were debriefed.

In the UAE sample, participants were told that the experiment investigated how easily people can read emotions on other people’s faces. They learned that they would see several videos of women talking about their experiences and that we would examine whether the quality of the videos and the visibility of the face influences how good people are in reading emotions. They further learned that they would watch the videos on silent, to investigate people’s abilities to decode emotions from non-verbal communication alone. Participants were told that the task might feel a bit difficult and were asked to make their best guess as to what each woman was feeling. As with the Dutch sample, all participants first saw the target woman present a neutral story, then viewed the three emotional videos in a random order, completing the emotion recognition questionnaire after each video. Participants were again asked about decoding difficulty and anxiety along with several exit questions before being debriefed and thanked for their participation. Participants completed the study in an open research laboratory that housed 25 computers. Up to 8 participants completed the study at the same time, with at least one empty computer in between participants to prevent distraction. They received partial course credit for participation.

## Results

### Participant Background

Because we aimed to test the differences between two countries that vary in their exposure to symbols of Islamic identity, we first needed to investigate whether there were any substantial differences between the participants with different ethnic and national backgrounds within the UAE sample. We thus investigated whether the three UAE nationality groups (GCC, non-GCC, non-Arab) differed in whether they had ever worn niqab themselves. In total, 11 of the 104 women had ever worn a niqab. No significant differences were found between the three national groups *X*^2^ (2, 115) = 4.61, *p* = 0.10. We then examined whether there were differences based on participants’ experience with women wearing niqab. We first computed a new variable consisting of answers to questions inquiring whether they had family members or friends wearing a niqab (*r* = 0.45). We then conducted an ANOVA with the three groups as a factor and “exposure to niqabs” as dependent measure. We found no significant effect for Group, *F* (1, 142) = 0.42, *p* = 0.66. Because there were no significant differences within the UAE sample on exposure to or experience with niqab, we collapsed the groups and compared the complete UAE sample (*N* = 200) with the Dutch sample (*N* = 117). For sake of clarity we will refer to this factor as Country and to the two groups as Dutch and UAE, because of the location where the data were collected. This research has been approved by the local ethics committees of both universities.

### Anxiety and Decoding Difficulty

We first computed a MANOVA with Anxiety and Decoding Difficulty as dependent measures and Country (2: Dutch, UAE) and Face Coverage (2: Full, Covered (averaged across niqab and black bars conditions) as between-subjects factors.

*Anxiety*. We found a main effect of Country, *F* (1, 304) = 9.37, *p* = 0.002, partial η^2^ = 0.030, showing that overall, the Dutch participants (*M* = 3.41; *SD* = 0.09) felt less anxious than the UAE participants (*M* = 3.77; *SD* = 1.03). We also found a main effect of Face Coverage, *F* (1, 304) = 4.405, *p* = 0.013, partial η^2^ = 0.028, showing that participants from both countries felt more anxious in reaction to displays where the face was covered (*M* = 3.72; *SD* = 0.09) rather than not covered (M = 3.46; SD = 1.06). The interaction between Country and Face Coverage was not significant, *F* (1, 304) = 2.31, *p* = 0.10, partial η^2^ = 0.015.

*Decoding Difficulty*. We found a main effect of Country, *F* (1, 304) = 13.19, *p* < 0.0001, partial η^2^ = 0.042, showing that participants from the UAE found it more difficult to rate emotions than the Dutch (UAE: *M* = 4.29; *SD* = 0.058; Dutch: *M* = 3.95; *SD* = 0.073. There were no other main or interaction effects.

### Emotion-Congruent Intensity Ratings

The data were analyzed in a linear mixed multilevel model with trials nested within participants. With this statistical method it is possible to include all sampled data-points in the analysis without the necessity to average over trials. That way, all variance in the data is maintained and with the possibility to include fixed and random factors, the statistical model can be set up in a way so that it most optimally explains this variance (see Table 1 for the means and standard deviations per condition and Table 2 for the statistical model).

**TABLE 1:** Descriptives.

**Emotion display**	**Face coverage**	**UAE**		**Dutch**	
		**Mean**	**SD**	**Mean**	**SD**
Anger	Full Face	4.768	1.687	5.065	1.299
	Black bar	3.000	2.156	3.573	2.170
	Niqab	2.871	2.080	2.895	1.982
Happy	Full Face	4.598	1.886	5.500	1.330
	Black bar	3.040	1.810	3.917	1.709
	Niqab	2.661	1.830	3.023	1.903
Shame	Full Face	4.089	2.034	4.837	1.775
	Black bar	4.774	1.983	4.542	1.794
	Niqab	4.460	1.914	4.535	1.720

*Emotion-congruent intensity ratings. Means are followed by the standard deviation (SD).*

**TABLE 2:** Emotion-congruent intensity ratings.

**Type III tests of fixed effects**							

**Source**			**df**		**F**		**Sig.**
Intercept			8, 1.893		45.830		0.000
Face coverage (y/n)			1, 1.893		138.937		0.000
Country			1, 1.893		18.995		0.000
Emotion display			1, 1.893		11.762		0.000
Face coverage * Emotion display			2, 1.893		54.654		0.000
Country * Emotion display			2, 1.893		3.648		0.026

**Estimates of covariance parameters**							
**Parameter**		**Estimate**	**Std. error**	**Z**	**Sig.**	**95% confidence interval**	
						**Lower bound**	**Upper bound**

Variance		3.391	0.121	28.098	0.000	3.163	3.636
Intercept [subject = id]	Variance	0.121	0.058	2.072	0.038	0.047	0.311

*Final model with intensity rating as the dependent variable. Face coverage was either “Not Covered” or “Covered.”*

In a first analysis, we included the following fixed predictors in the model: Face Coverage (2: Full, Covered (niqab and black bars condition were pooled), Country (2: Dutch, UAE), Emotion Display (Anger, Shame, Happiness). The dependent measures were the congruent emotion ratings, namely the intensity of anger rated after the Anger Display, the intensity of shame rated after the Shame Display and the intensity of Happiness rated after the Happiness Display. We started with a model that included only fixed factors. In order to get the most parsimonious model with the best fit, non-significant effects were removed one by one, starting with the higher-order interactions. Via likelihood ratio tests, we verified whether the removal of a non-significant factor improved the fit of the model or significantly decreased model fit. In the latter case the factor was retained in the model, otherwise it was excluded. After specifying the fixed effects, model building proceeded with statistical tests of the variances of the random effects (West et al., 2006). A random intercept for each individual subject was included.

**FIGURE 2:**
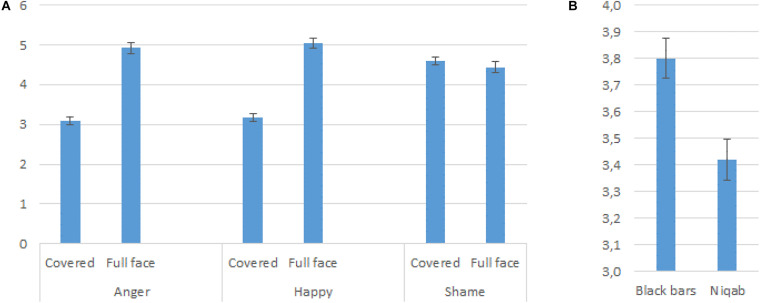
Emotion congruent intensity ratings. **(A)** Predicted means showing an interaction between emotion display and face coverage; **(B)** Main effect of face coverage type showing that participants rated women wearing a niqab as expressing emotions less intensely compared to women whose faces were partly covered by black bars.

*Full face versus Covered Face.* We observed a main effect of Face Coverage (*p* < 0.001) showing that, as expected, Face Coverage led to less intense emotion ratings. Furthermore, the UAE gave lower intensity ratings than the Dutch, as demonstrated by a main effect of Country (*p* < 0.001). A third main effect of Emotion Display showed that intensity scores were higher for shame compared to anger and happiness (see Table 2). In addition, the interaction between Face Coverage and Emotion Display (*p* < 0.001) showed that happiness and anger intensity ratings decreased significantly when faces were covered rather than uncovered (see Figure 2A).

We also found an interaction between Country and Emotion Display (*p* < 0.001) showing that the two groups had similar intensity ratings for shame but that the UAE participants gave lower happiness and anger ratings than the Dutch.

*Niqab versus Black Bars.* In order to test whether there was a specific effect of face coverage by a niqab versus black bars, we ran the same model as above, but by excluding the full face condition and including the niqab and black bars condition as Face Coverage Types. Apart from the earlier reported effect, a main effect of Face Coverage Type was observed (see Figure 2B). Independent of participants’ country of origin and independent of the emotion that was displayed, participants rated the emotions as less intense when the target face was covered by a niqab compared to black bars, thus providing some support for the effect of contextual cues (see Table 3 for the final model).

**TABLE 3:** Emotion-congruent intensity ratings.

**Type III tests of fixed effects**						
**Source**			**Numerator df**		**F**	**Sig.**
Intercept			6		32.764	0.000
Country			1		6.765	0.009
Emotion display			2		78.153	0.000
Face coverage			1		12.459	0.000
Country * Emotion display			2		3.667	0.026

**Estimates of covariance parameters**						
**Parameter**	**Estimate**	**Std. error**	**Z**	**Sig.**	**95% confidence interval**	
					**Lower bound**	**Upper bound**

Residual	3.744	0.148	25.328	0.000	3.465	4.045
Intercept [subject = id]: Variance components: This parameter is redundant						

*Final model with intensity rating as the dependent variable. Face coverage was either “Niqab” or “Black bar.”*

The current study focuses on the congruent ratings only. For completeness, we also included confusion matrices. Because of the abundance of possible statistical comparisons here, we decided to refrain from those altogether. The matrixes are to be interpreted with caution but may inspire future research (see Supplementary Table 1).

## Discussion

The present study tested differences in emotion intensity in covered and uncovered faces between a sample from the UAE and the Netherlands. Participants from both countries felt more anxious at seeing covered faces compared to fully visible faces. In contrast to what we expected, this effect was not enhanced in the Dutch. Across both samples, we replicated our earlier finding that covering the face – with either a niqab or with black bars – leads to the perception of less intense happiness (Fischer et al., 2012). We observed a similar, somewhat weaker pattern for the anger ratings. Specifically, these were also lower when the face was covered as compared to fully visible, a finding which is in contrast with our previous study where no effect was observed for anger (*p* = 0.50). An explanation may be that compared to expressions of shame, which relies mostly on eye cues, the mouth is important for recognizing happiness and, to a lesser extent, anger. Because the cues of these expressions were hidden, participants “saw” less happiness and anger, a finding consistent with the *Expressive Cues Hypothesis*. We also observed that participants rated women wearing a niqab as less emotional than women whose faces were covered by black bars, a finding speaking for the *Contextual Cues Hypothesis*. However, further research needs to confirm this finding and rule out potential confounding factors such as differences between the niqab and black bars condition underly this effect. Thus, although the general coverage of the mouth has profound effects on the perception of happiness and anger, we found some evidence suggesting that on top of that, niqab may affect emotion perception not only in Western people who are exposed a lot to negativism around the Islam, but also in people who are exposed to more symbols of Islamic identity. However, rather than such symbols reducing perceptions of positive emotions or increasing perceptions of negative ones, in this study, wearing a niqab reduced the perception of both. Perhaps covered women, perceived as more conservative or submissive, are expected to express less emotion overall. We propose that these two factors, face coverage and contextual cues may have complementary effects on face perception but that further research is needed to pull them apart and also, to further investigate the potential role of anxiety underlying the observed effects.

One difference between the two samples that should be noted is that the UAE sample was less comfortable with the task at hand: they reported more difficulty with emotion perception overall and they reported more anxiety. We do not exactly know how to explain this difference. It may be due to differences in experiences with such tasks, or with a more general difference in response style that may also explain the higher overall emotional intensity perceived by the UAE sample. However, this conclusion is speculative and not clearly supported on the basis of the present data alone.

One limitation of the present study is that the women portraying these emotions in the different face conditions were Dutch, which may also explain why participants from the UAE indicated to have more difficulties decoding the emotions compared to Dutch participants and also generally gave lower intensity ratings. However, it should be noted that the UAE is highly nationally diverse, with 89% of the resident population holding foreign passports. Thus, although the women depicted in our stimulus materials held Dutch nationality, we do not expect their appearance to be judged as highly unusual or unexpected. Indeed, we specifically chose women with dark hair and eyes who look quite similar to many Arab nationals. As far as we are aware, there is no existing dataset that categorize how and how intensely people within the UAE compared to other countries express emotions. That said, we do know that in honor culture contexts, including the UAE, both anger and shame are afforded (Boiger et al., 2014; Maitner et al., 2017), and thus we may expect people to clearly express them. If that is the case, people should also be able to recognize such expressions. That is, unlike in East Asia, we would not expect people to actively mask anger in the UAE. Still, future research should include a greater variety of models from different countries or ethnic backgrounds to test for generalizability of our findings.

We think that one strength of this study is that the videos that were used are ecologically valid, because they are dynamic and reflect real life observations in that they showed a talking woman expressing an emotion. These stimuli may also be responsible for the differences that were found with respect to other studies where stronger effects of contextual cues were found. The length of the video may have provided more expressive cues than the very short presentations of photos displaying an emotion in other studies (e.g., Kret and de Gelder, 2012).

Finally, we think that this research shows that in some circumstances the mere invisibility of facial cues, in this case a smiling or angry mouth, is sufficient to elicit different inferences from emotional experiences in target people. This is not to say that contextual cues play no role at all. In the current study, there was a tendency for participants to rate women wearing a niqab as less emotional than women whose faces were covered by black bars, a finding that is partly consistent with previous research which supported the contextual cue hypothesis (Kret and de Gelder, 2012; Kret and Fischer, 2018; Liedtke et al., 2018). The findings do not replicate completely, possibly because the effect of contextual cues in addition to expressive cues may depend on the amount of information that is available. For example, making fast judgments on the basis of a 100-millisecond exposure to a photo, in accordance with the task that was used in these studies, is likely to motivate the usage of different cues than inferences based on 1-min long dynamic videos, as was the case in the present study.

An open question is whether a niqab affected emotional perception differently for Dutch participants compared to UAE participants. For Dutch participants, stereotypes might have affected perception, for UAE participants, stereotypes may have mattered less, or differently. On the other hand, at least some of our UAE participants might be more used to reading emotions from women that wear a niqab – and thus this experience might affect overall perception. In a previous study in a Western sample (Kret and de Gelder, 2012) we looked at potential effects of stereotypes on the perception of emotions from faces covered by a niqab. However, we found very little variance in the questionnaire data and suspect that most people filled in the questionnaire in a socially desirable way. This is also seen in studies of the implicit association test (IAT) or other implicit measures, where implicit biases do not always correlate with explicit ones (for a meta-analysis on this topic, see Hofmann et al., 2005). In a future study, it would be interesting to replicate this study and correlate the results with different implicit measures illuminating biases associated with niqab.

The current study shows that the covering of the lower part of the face alters the perception of happiness and, to a lesser extent, anger. Even though this effect – reflecting reduced expressive cues – is likely to be a universal phenomenon, it may be amplified when negatively evaluated contextual cues are provided. This work also has important implications for the current obligation to wear medical masks to prevent the spread of Covid-19. Ironically, whereas laws may prohibit wearing a niqab at the moment, governments may require medical or homemade masks. In a public health crisis, the decoding difficulty that comes with facial covering is deemed tolerable, referring to preventing the spread of disease. However in absence of such a health crisis, governments, courtrooms, airports, and other public spaces may take into account the importance of seeing a person’s full face to allow for better understanding of a person’s emotions.

Future research could make a direct comparison between the effects of different types of naturalistic face coverage. We suspect that any covered faces will be perceived as less emotional, though stereotypes people associated with specific facial coverings may moderate such effects. Given that masks have been politicized in some parts of the world, and are thus seen as positive or negative cues in different contexts, it is possible that individual differences in the way people evaluate such cues will be important to how wearers are perceived and evaluated. We suspect that all covered faces will be perceived as less emotional, though we cannot exclude that in some contexts stereotypes people associated with specific facial coverings may moderate such effects. The current study may inform the debate on wearing a niqab or other face coverage in public places, as doing so may indeed lead to misinterpretation and miscommunication, thereby impacting social interactions. Such uncomfortable interactions may reflect the absence of a visible expressive mouth, but may be amplified by activated stereotypes or negative feelings.

## Data Availability Statement

The raw data, SPSS file, scripts, and other materials are all available on Dataverse.nl.

## Ethics Statement

The studies involving human participants were reviewed and approved by University of Amsterdam and American University of Sharjah. The patients/participants provided their written informed consent to participate in this study.

## Author Contributions

AF and AM designed the study and collected data. MK analyzed the data. All authors contributed to the interpretation of the results and to the writing.

## Conflict of Interest

The authors declare that the research was conducted in the absence of any commercial or financial relationships that could be construed as a potential conflict of interest.

## Footnotes

1 In the Dutch sample, the following other emotions were asked as well: contempt, irritation, embarrassment, anxiety, nervousness, cheerfulness, and joyfulness. For brevity of the questionnaire these emotion terms were not presented in the UAE sample. In the UAE sample we also asked questions about stereotypes, display rules, attitudes and expectancies. We do not report these measures as they were not included in the Dutch study.

## Supplementary Material

Supplementary Table 1 All ratings (congruent and incongruent).
